# The Impact of Spiritual Leadership on Nurses’ Spiritual Care Behavior: A Cross-Sectional Study of Chinese Nurses

**DOI:** 10.3390/healthcare14121634

**Published:** 2026-06-09

**Authors:** Yuqian Sun, Siyu Chen, Zhongliang Li, Qiqi Peng, Xuan Li, Yijia Zhao, Tingxi Zhou, Wenchi Zou, Xu Hong

**Affiliations:** 1 School of Business, Macau University of Science and Technology, Macau 999078, China; 2 Baoji Third Hospital, Baoji 721000, China; 3 The Third Affiliated Hospital of Guangzhou Medical University, Guangzhou 510150, China; 4 School of Labor and Employment Relations, University of Illinois Urbana-Champaign, Champaign, IL 61820, USA

**Keywords:** spiritual leadership, career calling, spiritual care behavior, empathy, nurse

## Abstract

**Background/Objectives**: Spiritual care is core to holistic patient care, yet a persistent implementation gap exists in Chinese hospitals. This study examines the association between spiritual leadership and nurses’ spiritual care behavior, with career calling as mediator and empathy as moderator. **Methods**: A cross-sectional design was adopted. Data were collected from 323 frontline nurses in 10 public hospitals across five provinces in China from June to September 2025 using validated Likert scales. Analyses included confirmatory factor analysis, hierarchical regression, and a second-stage moderated mediation model with 5000 bootstrap resamples using SPSS 26.0 and Mplus 8.3. **Results**: Spiritual leadership was positively associated with both nurses’ spiritual care behavior and career calling. Career calling partially mediated the relationship between spiritual leadership and nurses’ spiritual care behavior. Furthermore, empathy significantly strengthened the positive association between career calling and spiritual care behavior, and amplified the indirect effect of spiritual leadership on nurses’ spiritual care behavior via career calling. **Conclusions**: Spiritual leadership, career calling, and empathy are key factors associated with nurses’ spiritual care delivery. Targeted interventions for these factors can bridge the spiritual care implementation gap and enhance holistic patient care.

## 1. Introduction

### 1.1. Background

Although spiritual care is an indispensable part of the healthcare system [1], it has historically been marginalized [2]. The World Health Organization (WHO) not only incorporates spirituality as one of the four core dimensions of health but also clearly indicates that spiritual care is a key factor in enhancing the overall quality of life of patients [3]. At present, the global nursing academic community generally recognizes that spiritual care is the core pillar of holistic patient-centered care. Its implementation directly affects patients’ quality of life, treatment outcomes and positive perception of illness, serving as a key evaluation indicator of high-quality nursing services [4]. However, there is still an obvious “awareness–practice gap” in clinical practice. Research has reported that although over 90% of nurses worldwide recognize the importance of spiritual care [2], most patients still do not have access to such services [5]. This gap is particularly pronounced in Asian healthcare contexts [6], with recent studies in mainland China showing a stark awareness–practice gap in clinical spiritual care: while the vast majority of frontline nurses recognize the necessity of spiritual care, only 8.9% of them report having consistently delivered this care in daily clinical practice [7]. Further evidence shows that Chinese community nurses’ spiritual care competence is generally insufficient, highlighting the urgency of exploring motivational and organizational drivers of spiritual care behavior [7]. This awareness–practice gap not only undermines patients’ right to spiritual care but also violates the basic principle of patient-centered care and high-quality healthcare. Therefore, it is critical to explore how hospital managers can motivate nurses to incorporate spiritual care for patients into routine clinical practice, especially in the Asian healthcare context.

While earlier work has established that nursing education and capacity building remain essential to providing spiritual care, modern research is increasingly highlighting how nurses’ personal attributes play an important correlational role in how they carry out spiritual care in practice [8]. Research on nurses’ spiritual well-being, attitudes toward spiritual health, and antecedent factors including religious beliefs, professional responsibility, and transcendent consciousness has deepened our understanding of the mechanisms underlying spiritual care implementation [9]. It is worth noting that most existing studies overlook a fundamental theoretical driver of sustained behavior: nurses’ intrinsic motivation. According to the self-determination theory (SDT), the generation and maintenance of nurses’ altruistic behaviors need to meet three basic psychological needs: autonomy, competence, and relatedness [10]. As a manifestation of intrinsic motivation, the sense of career calling promotes continuous behavior by internalizing the meaning of work into the core component of self-identification [11]. Recent research has indicated that management, leadership, personal resources and job resources are all associated with nurses’ spiritual care competence [12,13]. However, the core motivational mechanism underlying these factors remains insufficiently explored. Prior research has found that this meaning-making process enables nurses to internalize patient well-being as a core personal responsibility, inspiring them to proactively undertake non-mandatory responsibilities including providing spiritual care and exhibiting higher levels of caregiving behavior [14]. At the same time, this career orientation is closely related to higher career resilience and continuous altruistic motivation [15]. Collectively, these findings suggest that the supportive motivational resources provided by a sense of career calling may be a key antecedent for nurses to overcome practical barriers and deliver consistent spiritual care.

Accordingly, this study seeks to identify the contextual factors that can cultivate nurses’ sense of career calling. Prior research has revealed that spiritual leadership systematically shapes nurses’ motivational structure through three interrelated dimensions: vision, faith/hope, and altruism [16,17]. A 2026 systematic review of spiritual leadership in healthcare settings further confirmed that Fry’s spiritual leadership theory is the most widely applied theoretical framework in the nursing field. Meanwhile, numerous empirical studies have verified its positive association with nurses’ work attitudes, psychological status, and caring behaviors [18]. This aligns perfectly with all three core psychological needs proposed by SDT. For example, when spiritual leadership combines nurses’ daily routine caring work with transcendental values, it will trigger the sequential mechanism of “spiritual leadership → positive psychological state (e.g., career calling) → extra-role behavior (e.g., spiritual care)” [10,17,19]. Therefore, this study posits that spiritual leadership can act as a crucial contextual antecedent, which enhances nurses’ career calling and thus bridges the awareness–practice gap in spiritual care. Specifically, spiritual leadership emphasizes shared vision, altruistic love, and meaning construction among nurses. This can improve nurses’ awareness that their work is not only a profession, but also a career calling, thus inspiring them to consistently fulfill their spiritual care responsibilities.

Furthermore, prior research has identified nurses’ individual characteristics as key boundary conditions that shape the translation of nurses’ cognition into clinical behaviors [20]. We choose empathy as the key boundary condition for two theoretical reasons. Empathy is a core clinical competency that helps nurses accurately perceive and respond to patients’ unspoken spiritual needs. It is a necessary prerequisite for effective spiritual care. Communication skills and spiritual literacy alone cannot fully replace empathy [21]. Second, empathy directly bridges the gap between intrinsic motivation and action by translating internalized values into targeted behavioral responses. We therefore propose that nurses’ empathy moderates the relationship between career calling and spiritual care behavior.

While existing studies have explored predictors of nurses’ spiritual care behavior, three key research gaps remain to be addressed [7]. First, most research focuses on individual-level factors or the direct effect of leadership, with few constructing an integrated framework linking contextual leadership, intrinsic motivation, and behavioral outcomes, leaving the psychological mechanism linking spiritual leadership to spiritual care underexplored. Second, prior work has not clearly distinguished the stages of motivation formation and motivation transformation based on SDT, leading to ambiguity around the precise boundary role of empathy in translating intrinsic motivation into clinical behavior. Third, most evidence comes from Western healthcare settings, with limited large-sample empirical research in Chinese public hospitals, where the clinical and cultural context differs notably from Western contexts.

**Figure 1:**
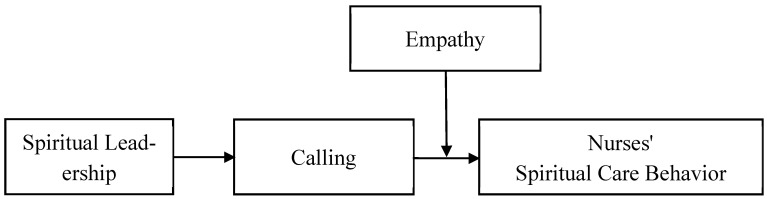
Hypothesized Model.

To address these gaps, this study develops a moderated mediation model based on SDT and Spiritual Leadership Theory, we explore how spiritual leadership is associated with nurses’ spiritual care behavior via career calling, and the boundary role of empathy in this pathway. This study complements existing literature by clarifying the motivational mechanism of spiritual care behavior, identifying the specific role of empathy, and providing localized evidence for Chinese clinical settings. The conceptual framework is shown in Figure 1.

### 1.2. Theory and Hypotheses

#### 1.2.1. Spiritual Leadership and Spiritual Care Behavior

Spiritual care covers the systematic nursing interventions provided by nursing staff in clinical practice, the purpose of which is to meet the religious and existential needs of patients, manifested in respecting patients’ beliefs and values, listening carefully to spiritual concerns, and promoting meaning construction. The effective provision of spiritual care requires individuals to integrate cognitive ability, emotional ability and technical proficiency into daily work practice, helping patients maintain inner balance during health crises [22]. The successful implementation of spiritual care depends on nurses’ competencies and a comprehensive hospital support system [8].

Based on the core logic of SDT, the sustained and voluntary demonstration of nurses’ spiritual care, as a typical extra-role altruistic behavior, must be premised on the satisfaction of three core basic psychological needs: autonomy, competence, and relatedness [10]. Only when these three needs are continuously met can nurses form stable intrinsic motivation, and then translate this motivation into long-term consistent spiritual care behavior, rather than temporary passive compliance [23]. Spiritual Leadership Theory further proposes that spiritual leaders, as the core providers of organizational supportive resources in clinical settings [24], can systematically satisfy nurses’ three basic psychological needs through three interlocking dimensions: vision, faith/hope, and altruism [17]. This creates a complete theoretical causal chain from contextual leadership support to intrinsic motivation formation, and finally to clinical behavioral output, which can fully explain why spiritual leadership can stably associated with nurses’ spiritual care behavior.

Spiritual leaders influence their followers through vision, altruism, and faith/hope [16]. The leader’s explanation of the shared vision, demonstration of sincere care, and expression of optimistic belief are closely associated with the intrinsic motivation of nurses to provide spiritual care [19]. The latest systematic review of spiritual leadership in healthcare also found that the vision and altruistic love dimensions of spiritual leadership can effectively create a supportive organizational climate, reduce nurses’ emotional exhaustion, and enhance their willingness to implement patient-centered care [18]. Spiritual leadership promotes the integration of the hospital’s mission and personal values by developing a meaning-driven system that transcends material rewards [25]. When spiritual leaders convey a vision centered on alleviating patient suffering and upholding human dignity, nurses are more likely to perceive their work as meaningful beyond routine care responsibilities [14]. This fit of personal values and hospital mission fully satisfies nurses’ need for autonomy: they no longer perceive spiritual care as an additional mandatory task, but as a voluntary behavior consistent with their own values, stimulating their internal motivation to actively engage in spiritual care [26].

In addition, spiritual leaders can clarify compassionate nursing concepts by providing resources, training and encouragement to nurses [27,28]. This helps nurses develop the necessary skills and confidence to effectively meet the spiritual needs of patients. When nurses believe that they have the ability to respond effectively to the spiritual needs of patients, they will be more motivated to develop the necessary skills and knowledge and apply them in practice [29]. This increased sense of competence empowers nurses to take the initiative in spiritual care.

Finally, the altruistic love dimension of spiritual leadership nurtures a sense of relatedness among nurses [17]. By demonstrating care and support for nurses, leaders create a trusting and safe environment [30]. This sense of security and team belonging fully satisfies nurses’ need for relatedness, making them more willing to invest extra energy in non-mandatory spiritual care work, rather than just completing the minimum required clinical tasks. Taken together, spiritual leadership should be positively associated with nurses’ spiritual care behavior by systematically satisfying all three core psychological needs proposed by self-determination theory. Thus, we hypothesize the following:

**H1.**  *Spiritual leadership is positively associated with nurses’ spiritual care behavior*.

#### 1.2.2. Spiritual Leadership and Career Calling

In nursing, career calling means a transcendent and internal professional attitude. It features a strong recognition of nursing’s altruistic values, a sense of purpose in clinical work, deep professional identity, and viewing nursing as a life mission consistent with personal core values [31,32]. SDT points out that the formation and deepening of highly internalized career calling, a key manifestation of intrinsic motivation, is closely tied to the continuous satisfaction of autonomy, competence and relatedness needs. When these three needs are consistently met, nurses are more able to internalize the altruistic mission of nursing into their core self-identity, rather than only regarding nursing as a means of making a living, which forms the core theoretical premise for the generation and strengthening of career calling [10,33].

First, spiritual leadership supports nurses’ career calling by satisfying their need for autonomy. Spiritual leaders connect nursing work to the transcendent altruistic goal of safeguarding human dignity and improving patient health, instead of viewing it as simple task fulfillment. They also respect nurses’ professional autonomy in clinical decisions and avoid controlling behaviors [14]. This autonomy support enables nurses to freely internalize the altruistic values of nursing into their own core self-concept, which is the core prerequisite for regarding nursing as an inner life calling, rather than a passive job requirement [27]. Secondly, spiritual leadership enhances nurses’ career calling by meeting the competence needs of nurses. Spiritual leaders can help nurses cope with clinical challenges and cultivate a sense of professional self-efficacy [17,27]. The sense of competence satisfaction can continuously strengthen nurses’ belief in their ability to fulfill the altruistic calling of nursing, making them more firmly identify with the mission of the profession, thus deepening their sense of career calling [34]. Third, spiritual leadership enriches the core value of nurses’ profession by meeting the needs of nurses’ relatedness. Spiritual leaders care about the physical and mental health of nurses, create a team atmosphere of mutual trust and tolerance, and promote the emotional connection between nurses and patients [29,35]. Relatedness satisfaction nurtures prosocial values that are consistent with nursing’s inherent altruism. It encourages nurses to deeply identify with the mission of nursing and turn it into personal life pursuits [36]. Collectively, spiritual leadership should be positively associated with nurses’ career calling by systematically satisfying all three core psychological needs. The latest empirical studies also confirm that spiritual leadership significantly and positively associated with career calling [37]. Thus, we hypothesize the following:

**H2.**  *Spiritual leadership is positively associated with nurses’ career calling*.

#### 1.2.3. Career Calling and Spiritual Care Behavior

For nurses with a strong sense of career calling, caring for their patients is not merely a job but a deeply fulfilling mission that enables them to attain a profound sense of purpose and professional accomplishment [38]. Consistent with the core tenets of SDT, career calling, as a deeply internalized form of intrinsic motivation [39], fosters the sustained and stable fulfillment of nurses’ three basic psychological needs, in turn motivating them to voluntarily engage in behaviors consistent with their core professional values. This forms the core theoretical mechanism linking career calling to spiritual care behavior. Specifically, career calling satisfies nurses’ need for autonomy. Nurses with a strong sense of autonomy tend to act in line with their own inner values. Career calling strengthens nurses’ autonomy, allowing them to prioritize spiritual care in clinical practice without external pressure [25].

Career calling fulfills the need for competence by encouraging active skill improvement. Nurses motivated by career calling are more willing to attend spiritual care training, reflect on their clinical practice, and build the professional skills required to meet patients’ complex spiritual needs [34]. This improved competence further boosts their confidence in delivering spiritual care and forms a positive feedback loop.

The career calling of nurses emphasizes the establishment of pro-social interpersonal ties with patients and respect for the human nature of patients [40]. It focuses on the positive interpersonal relationship between nurses and patients. Nurses with a sense of career calling can establish a strong connection with patients and regard them as unique individuals, not just clinical cases [25]. This strengthens the nurse–patient bond and fosters relational caring, a core antecedent of spiritual care motivation [20,30]. Taken together, career calling as an internalized intrinsic motivation should be directly associated with nurses’ spiritual care behavior. Thus, we hypothesize the following:

**H3.**  *Career calling is positively associated with nurses’ spiritual care behavior*.

Based on the above theoretical derivation, we further construct the mediating mechanism of career calling, which is fully rooted in SDT’s “contextual support → intrinsic motivation formation → behavioral output” core theoretical framework [18,41]. Spiritual leadership, as the organizational contextual antecedent, cannot directly translate into nurses’ clinical behavior; it must first shape nurses’ intrinsic motivation by satisfying their three basic psychological needs, and then is linked to their behavioral changes. Career calling, as a deeply internalized form of nurses’ internalized intrinsic motivation, is exactly the core mediating bridge between contextual leadership support and final clinical behavioral output. In sum, we hypothesize the following:

**H4.**  *Career calling mediates the relationship between spiritual leadership and nurses’ spiritual care behavior*.

#### 1.2.4. The Moderating Effect of Empathy

When nurses regard work as a career calling, they will be driven by inner motivation to provide care in line with their own values and mission. However, the extent to which this motivation can be transformed into actual spiritual care depends on their ability to accurately understand and respond to the patient’s emotional state—especially their empathy. Davis [42] believes that empathy consists of two dimensions: cognitive empathy, which reflects the ability to accurately understand the views and emotions of others; and affective empathy, which represents the tendency to experience the emotions of others. As a stable clinical competency formed through long-term professional education and practice, empathy not only affects the depth of nurses’ perception of patients’ needs [43], but also determines the efficiency of translating intrinsic motivation into observable clinical behavior.

Consistent with SDT’s core causal sequence, the model can be divided into two theoretically distinct stages: the motivation formation stage (spiritual leadership → career calling) and the motivation translation stage (career calling → spiritual care behavior). Spiritual leadership shapes career calling by satisfying nurses’ basic psychological needs, a process shaped by organizational contextual factors rather than individual perceptual abilities. In contrast, empathy is directly associated with whether nurses can effectively translate their formed intrinsic motivation into actual spiritual care behavior. Therefore, empathy acts as a boundary condition specifically in the second stage of the mediation model.

Nurses with high empathy are more likely to turn their sense of career calling into spiritual care behavior [31]. Nurses with a high level of empathy can deeply understand the inner needs of patients. For example, they can deeply understand patients’ inner needs, such as fears, anxieties, and yearnings for meaning and connection during illness. In this situation, nurses’ strong sense of career calling makes them try to alleviate patients’ suffering and demonstrate more spiritual care actions.

Cognitive empathy serves as a crucial antecedent for nurses’ behavioral tendencies [44]. Highly empathetic nurses can distinguish non-physical pain other than the patient’s superficial symptoms, and this accurate perception amplifies the altruistic motivation shaped by career calling, inspiring them to meet patients’ deeper spiritual needs. For instance, a nurse with high empathy who views their work as a calling is more likely to notice a patient’s unspoken anxiety about existential issues or a desire for connection, spiritual distress, and proactively respond through active listening and deep conversations about their existential concerns, providing emotional support, and facilitating spiritual support [26,45]. Conversely, nurses with a sense of career calling but lower empathy might struggle to effectively manifest their calling in spiritual care practice. This is because they may encounter difficulties in fully comprehending or relating to patients’ spiritual experiences or needs. This situation will restrict the transformation of their intrinsic motivation into the actual provision of spiritual care [14,25]. Prior research has confirmed that empathy plays an important role in the process of transforming nurses’ internal care motivation into actual clinical behaviors [46]. Drawing on this two-stage framework, nurses with higher empathy should be better able to convert their career calling into patient-centered spiritual care. Therefore, it is reasonable to hypothesize that nurses with high empathy can better transform their career calling into patient-centered spiritual care. Accordingly, we hypothesize:

**H5.**  *Empathy moderates the relationship between career calling and spiritual care behavior such that the relationship between career calling and spiritual care behavior is strengthened when nurses’ empathy is higher than when it is low*.

The aforementioned hypotheses collectively suggest a second-stage moderated mediation model. Specifically, since empathy positively moderates the strength of the relationship between career calling and spiritual care behavior, it will inevitably further moderate the strength of the entire indirect effect of spiritual leadership on spiritual care behavior via career calling:

**H6.**  *The indirect effect of spiritual leadership on nurses’ spiritual care behavior via career calling is moderated by empathy, such that the indirect effect is stronger for nurses with higher levels of empathy*.

## 2. Materials and Methods

### 2.1. Procedure

This study employed convenience and snowball sampling to recruit frontline nurses, consistent with established practices for this hard-to-reach population [47,48]. This strategy was selected for its practical feasibility in accessing nurses with heavy workloads and rotating shifts [47,49,50], its ability to facilitate honest responses to sensitive questions by building trust through existing relationships [47], and its potential to enhance sample heterogeneity across clinical settings [51]. Our single-source self-report design also avoids the risk of fabricated informant ratings, a documented limitation of multisource snowball research [49].

The data were collected from state-owned hospitals in the Chinese provinces of Guangdong, Shanxi, Jilin, Anhui, and Heilongjiang from June to September 2025. To minimize sampling bias, we implemented four rigorous control measures [49,52]: (1) Geographical diversification: Participants were recruited from eastern, central, western, and northeastern China to reduce network homogeneity bias. (2) Strict eligibility criteria: Only registered nurses with at least 1 year of direct clinical experience were included. Nurse managers, interns, and non-clinical nurses were excluded. (3) Anonymous participation: No identifiable information was collected to reduce social desirability bias. (4) High-quality data: A total of 323 valid responses were obtained, with a response rate of 76.9%, which is significantly higher than the average response rate in nursing research [53]. This multi-source, heterogeneous recruitment of nurses minimized the risk of initial sample homogeneity inherent in single-site convenience sampling [54].

We distributed the online questionnaire via WeChat using Wenjuanxing. An informed consent form was shown on the first page of the questionnaire. The nurses were informed about the confidentiality of their responses and their right to withdraw from the study at any stage without any negative impact on their current or future benefits. The study’s objectives, procedures, and ethical considerations were also briefly presented to the participants. If they agreed to participate in this study, they were required to click ‘consent’ before proceeding. The online questionnaire was completed anonymously. All survey items were set as mandatory responses, and there was no missing data in the final valid sample. Thus, no data imputation was performed.

### 2.2. Participants

A total of 420 online questionnaires were disseminated, and 323 valid responses were obtained, yielding a response rate of 76.9%. The average age of the valid participants was 35.7 years, with an average length of service of 13 years. Among them, 78.3% were female and 21.7% were male. Male nurses are mainly concentrated in ICU, emergency departments and surgery, which is consistent with the distribution characteristics of male nurses in Chinese clinical environment. The demographic and work-related data of all participants are shown in Table 1.

### 2.3. Measures

Participants responded to the survey questions using a Likert-type scale, with values ranging from 1 (strongly disagree) to 5 (strongly agree).

Spiritual leadership was evaluated using Fry and Cohen’s [16] scale. This scale encompasses three dimensions: vision, faith/hope, and altruism. An example item was “My direct leader has a vision statement that brings out the best in me.” The Cronbach’s α of this scale was 0.98.

Career calling was measured using the scale of Dobrow and Tosti-Kharas [11], which has been validated in the Chinese nursing context [34,35]. A sample item was “I am passionate about my medical/nursing work.” The Cronbach’s α of this scale was 0.91.

Spiritual care behavior was measured using the scale developed by Van Leeuwen et al. [22]. A sample item was “I tailor care to patients’ spiritual needs in consultation with them.” The Cronbach’s α of this scale was also 0.91.

Empathy was evaluated using the Jefferson Scale of Empathy [55]. A sample item was “Understanding patients’ feelings influences treatment.” The Cronbach’s α of this scale was 0.90.

Control variables: Consistent with prior research, nurses’ age, gender, tenure, marital status, and monthly salary were included as control variables [56]. Job satisfaction was also included as a control variable, as prior research has shown it to be associated with nurses’ career calling and spiritual care behavior [31].

## 3. Results

Before hypothesis testing, we conducted a confirmatory factor analysis (CFA) to evaluate the measurement model. To construct our measurement model, we randomly created 3-item parcels for each of spiritual leadership, career calling, spiritual care behavior, and empathy. Model fit was evaluated according to the established criteria [57,58]. As shown in Table 2, the hypothesized four-factor model (spiritual leadership, career calling, spiritual care behavior, and empathy) exhibited excellent fit (χ^2^ = 84.875; *df* = 48; CFI = 0.988; TLI = 0.984; RMSEA = 0.049; SRMR = 0.027). These results confirm the discriminant validity of the four constructs and support the applicability of the measurement model.

To address potential common method bias as suggested by Podsakoff et al. [59], we employed an unmeasured latent method factor technique using Mplus. This model specified that all indicators load on both their theoretical factors and a general method factor. The bifactor model (χ^2^ = 65.362; df = 36; CFI = 0.990; RMSEA = 0.050; SRMR = 0.015) exhibited a negligible change in fit relative to the four-factor theoretical model (ΔCFI = 0.002), confirming that common method variance is not a critical issue in this study.

Furthermore, to identify the magnitude of multicollinearity, variance inflation factor (VIF) is a widely used metric. As a recently revised rule of thumb, the VIF values of the constructs in a model should be below 3.0 [60]. Results of the collinearity statistic showed that all variance inflation factor values ranged from 1.051 to 1.504, and tolerance values ranged from 0.665 to 0.952, confirming no serious multicollinearity problems [60]. We also conducted Harman’s single-factor test [61]. An exploratory factor analysis including all survey items revealed that a single factor accounted for 26.48% of the total variance before rotation, which is below the recommended threshold of 40%, suggesting there was no common method bias. Additionally, all HTMT (Heterotrait–Monotrait Ratio; [62]) values remained below the 0.85 threshold (maximum = 0.592), confirming adequate discriminant validity across constructs. The composite reliability (CR) values range from 0.899 to 0.966, exceeding the threshold benchmark of 0.70. The average variances extracted (AVE) ranged from 0.750 to 0.906, exceeding the cutoff point of 0.50 [63,64].

Table 3 presents the descriptive statistics, reliability estimates, and correlations of the control variables and main variables of the study. Cronbach’s α coefficients ranged from 0.90 to 0.98, indicating a high internal consistency reliability. A number of control variables demonstrated significant correlations with the primary variables under investigation. Age manifested a positive correlation with career calling (r = 0.11, *p* < 0.05), whereas gender was negatively correlated with career calling (r = −0.12, *p* < 0.05). Tenure correlated positively with both career calling (r = 0.20, *p* < 0.01) and spiritual care behavior (r = 0.14, *p* < 0.05). Similarly, marital status correlated positively with career calling (r = 0.20, *p* < 0.01) and spiritual care behavior (r = 0.16, *p* < 0.01). Monthly salary was negatively associated with spiritual leadership (r = −0.14, *p* < 0.05) but positively associated with spiritual care behavior (r = 0.15, *p* < 0.01). Job satisfaction demonstrated significant correlations with all four primary study variables.

Consistent with prior research and theoretical considerations, we controlled for age, gender, tenure, marital status, monthly salary, and job satisfaction in all subsequent analyses. Spiritual leadership demonstrated positive associations with both career calling (r = 0.56, *p* < 0.01) and nurses’ spiritual care behavior (r = 0.44, *p* < 0.01). Furthermore, career calling was positively associated with nurses’ spiritual care behavior (r = 0.52, *p* < 0.01), and spiritual care behavior was positively associated with nurses’ empathy (r = 0.32, *p* < 0.01).

### Hypothesis Testing

Hypotheses were tested using SPSS 26.0 with the PROCESS macro version 5.0 [65]. Table 4 shows that spiritual leadership exhibited a significant positive effect on both spiritual care behavior (b = 0.18, SE = 0.04, *p* < 0.001) and career calling (b = 0.40, SE = 0.04, *p* < 0.001). Furthermore, career calling exhibited a significant positive effect on spiritual care behavior (b = 0.31, SE = 0.05, *p* < 0.001). These results provide strong empirical support for Hypotheses 1, 2, and 3. Table 5 shows that spiritual leadership had an indirect effect on nurses’ spiritual care behavior via career calling (b = 0.14, SE = 0.03). The bootstrap analysis (95% confidence interval = [0.09, 0.19]) provided consistent evidence for the mediating effect, supporting Hypothesis 4. The interaction between empathy and career calling was significantly and positively associated with spiritual care behavior (b = 0.43, SE = 0.08, *p* < 0.001), supporting Hypothesis 5 and indicating that the relationship between career calling and spiritual care behavior is stronger for nurses with higher empathy compared to those with lower empathy.

**Figure 2:**
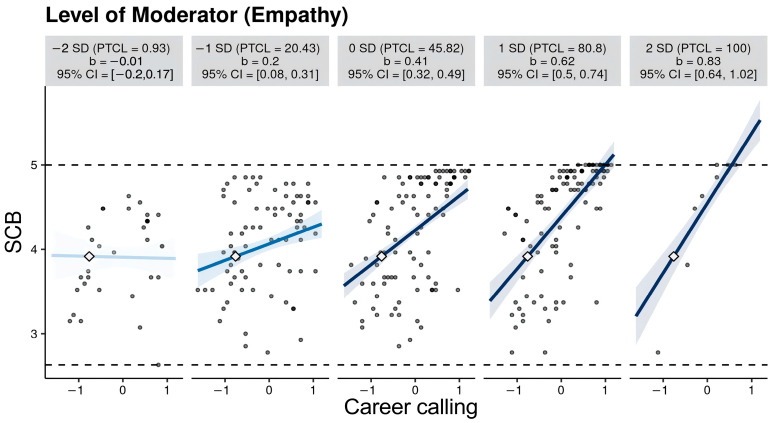
Simple slope with empathy ability as moderator. Two-way interaction of career calling and empathy in the prediction of spiritual care behavior Each graph illustrates the 95% confidence region (shaded area) calculated, the observed data (gray circles), and the upper and lower bounds of the outcome (dashed horizontal lines). The *x*-axis represents the level of career calling. CI = confidence interval; PTCL = percentile.

**Figure 3:**
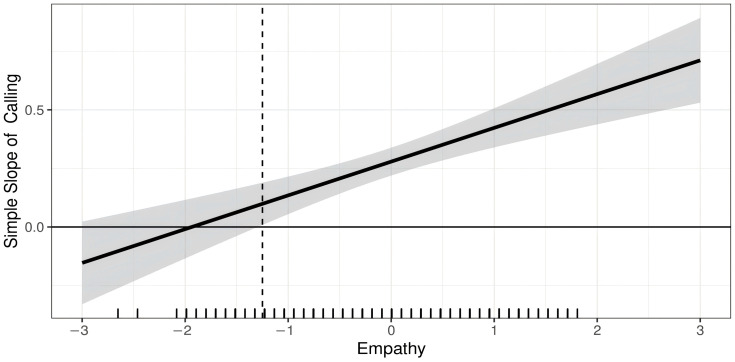
Marginal-effects plot with empathy as moderator. The shaded regions indicate 95% confidence intervals. A marginal rug is provided on the horizontal axis to indicate observed data across the displayed range of empathy. The vertical dashed lines represent the Johnson–Neyman values, which are the points on the empathy where the confidence intervals stop crossing zero, indicating the slopes in these regions are statistically significant and non-zero.

To further confirm the research results, simple slope analysis and post hoc tests were conducted to examine the moderation effect in detail [66]. As depicted in Figure 2, the analysis of the moderating effect uncovers the differential association between nurses’ sense of career calling and spiritual care behavior (SCB) at varying levels of empathy. The moderation analysis demonstrated that the effect of career calling on spiritual care behavior was not statistically significant at the low empathy level (−2SD; b = −0.01, 95% CI [−0.20, 0.17]). However, significant positive associations emerged at both mean and elevated empathy levels. Specifically, this relationship showed significant strength at a high level of empathy (+2SD; b = 0.83, 95% CI [0.64, 1.02]). As shown in Figure 3, the marginal effect analysis further elucidated that the simple slope of career calling on SCB attained statistical significance when empathy exceeded −1.25 standard deviations from the mean. It is worth noting that this region encompasses 87.93% of the observed empathy values. Together, these findings collectively suggest that the synergy between enhanced career calling and empathy significantly promotes the spiritual care behavior of the vast majority of nurses in the sample, further confirming Hypothesis 5. Clinically, this means that even nurses with strong career calling may fail to provide adequate spiritual care if they lack the ability to perceive patients’ unspoken spiritual needs, which explains why many well-intentioned nurses still struggle to implement spiritual care in practice. Notably, these findings differ from two recent studies that reported alternative causal directions and conceptual distinctions [67,68].

Regarding the moderated mediation Hypothesis (Hypothesis 6), we hypothesized that the positive correlation between career calling and spiritual care behavior is moderated by empathy, that is, nurses with high empathy will show a stronger correlation between career calling and spiritual care behavior. The analysis presented in Table 5 reveals that for nurses with high empathy, the indirect effect of spiritual leadership on spiritual care behavior via career calling is positive and statistically significant (B = 0.21, SE = 0.03, 95% CI [0.16, 0.27]). The calculated moderated mediation index is 0.17 (95% CI [0.11, 0.23]). These findings offer empirical support for Hypothesis 6.

In terms of practical significance, the effect sizes observed in this study are clinically meaningful. A one-standard-deviation increase in spiritual leadership was associated with a 0.18 standard deviation increase in spiritual care behavior, and career calling mediated approximately 44% of this total effect. Furthermore, nurses with high empathy (+1SD) exhibited a 75% stronger association between career calling and spiritual care behavior compared to those with average empathy.

## 4. Discussion

We have built a second-stage moderated mediation model based on spiritual leadership theory and self-determination theory. The model aims to explore the potential relational mechanism linking spiritual leadership to nurses’ spiritual care behavior. The empirical results provide robust empirical support for the proposed theoretical framework in the context of this study.

Spiritual leadership is significantly and positively correlated with career calling. The model also demonstrates significant associations with nurses’ spiritual care behavior. Career calling plays a mediating role between spiritual leadership and spiritual care behavior. Regarding the moderating factors of this mechanism, our analysis shows that nurses’ empathy moderates the relationship between career calling and spiritual care behavior. This study also demonstrates that empathy strengthens the indirect effect of spiritual leadership on spiritual care behavior via career calling. The study clarifies key factors associated with spiritual care behavior and makes important theoretical contributions. These findings provide actionable practical guidance for improving nurses’ spiritual care practice in clinical settings.

### 4.1. Theoretical Implications

This research makes several important contributions to the research of spiritual leadership, career calling, spiritual care behavior and nurses’ empathy. First of all, people are increasingly aware of the importance of spiritual care in health care. However, there is still a significant gap between the theoretical framework and practical application, and between cognitive understanding and clinical practice [4]. The existing research mainly focuses on the individual factors that affect the spiritual care behavior of nurses. This study promotes the development of this field by identifying spiritual leadership as a key situational factor. At the same time, the motivational relational mechanism linking leadership to clinical practice is revealed. Our research results provide practical insights on how to encourage nurses to participate more actively in spiritual care. Notably, inconsistent findings in prior literature warrant two key clarifications. First, Wang et al. [67] found that spiritual care perceptions positively predict empathy, indicating a potential bidirectional relationship. Second, Wu et al.’s [68] meta-analysis confirmed an association between empathy and spiritual care competence, but noted extreme heterogeneity and a well-documented gap between perceived ability and actual clinical effectiveness. Our study partially addresses this gap by examining how intrinsic motivation and empathic competence translate into observable spiritual care behavior.

Secondly, previous studies have explored different factors affecting spiritual care behavior, such as personal attitudes and mental health status [9]. However, these factors cannot fully explain why and how nurses continue to provide spiritual care to patients. Through SDT, this study explore how and why nurses’ sense of career calling is associated with their spiritual care behavior and also its role in the relationship between spiritual leadership and spiritual care. The study reveals a possible causal path: starting from leadership behavior, it is associated with the psychological needs of nurses, which in turn affects their professional nursing practice. The framework fully explains how spiritual leadership shapes the practice of spiritual care in the clinical environment and provides a theoretical basis for understanding this process.

Third, this study points out that empathy is an important personal quality of nurses, which is associated with the ability of nurses to provide spiritual care. Specifically, the moderating role of nurses’ empathy shows that the positive association between a sense of calling and spiritual care behavior is not the same for all nurses, and its effect depends on the level of empathy. This discovery promoted the research progress by distinguishing between two key stages of behavioral change—motivation formation (spiritual leadership → career calling) and motivation transformation (career calling → spiritual care behavior). The study identifies empathy as a key boundary condition, which determines whether the intrinsic motivation can be effectively transformed into clinical action. In addition, the moderated mediation analysis shows that spiritual leadership is indirectly associated with nurses’ spiritual care behavior by enhancing their sense of calling. When nurses have a high level of empathy, this indirect effect will be significantly enhanced. The results of this study not only deepen our understanding of the complex mechanism linking spiritual leadership to nurses’ behavior, but also provide new insights into how multiple factors interact to shape the professional practice of nurses.

### 4.2. Practical Implications

The moderated mediation model examined in this study provides potentially actionable strategies for nursing management and hospitals, especially for Chinese public hospitals and similar Asian clinical environments. Our effect size analysis shows that career calling explains 44% of the total effect of spiritual leadership on spiritual care behavior, and high empathy can strengthen this relationship by 75%, which provides a clear quantitative basis for the following intervention recommendations.

First of all, hospital nursing managers should develop their own spiritual leadership competencies as a core intervention. This can be achieved by clarifying the vision of common care related to the values of transcendental altruism, expressing concern for the well-being of nurses to build trust, and providing targeted resources and affirmation of mental care capabilities. This enables spiritual leaders to meet the psychological needs of nurses and enhance their sense of career calling [8,17]. These abilities should be incorporated into head nurse training and performance evaluation, with a specific focus on altruistic love and vision communication, delivered through quarterly 8 h modular training.

Secondly, institutions need targeted interventions to promote the cultivation of a sense of career calling in order to play their key role. Set up structured reflective practice activities, for example, 90 min spiritual care case conferences where nurses share experiences of finding meaning in clinical work. Also, set up a career calling-focused mentoring program. Pair junior nurses with senior nurses who are highly dedicated to the profession. These measures help nurses regard spiritual care as a core professional duty, rather than optional additional tasks [44]. Including this content in the continuous nursing education program can effectively cultivate the long-term motivation of nurses to implement spiritual care, particularly for nurses with less than 5 years of experience who are still forming their professional identity.

Third, systematic and continuous empathy training is crucial. This kind of training helps to turn the career calling of nurses into spiritual care behavior. Training should cultivate cognitive empathy and emotional empathy through interactive activities such as scenario role-playing and case discussion [44]. Cognitive empathy refers to identifying the patient’ s unspeakable spiritual needs, while emotional empathy requires consistent emotional management with professional standards. Empathy training should be targeted at the 12% of nurses with low empathy identified in our sample, focusing on cognitive empathy skills for identifying hidden spiritual distress, and integrated into daily clinical supervision rather than one-off workshops. In this way, nurses who provide empathy care can be rewarded accordingly.

Fourth, the nursing management department should formulate an overall plan. The program needs to coordinate the organization’s spiritual leadership building, career calling cultivation, ability-based empathy training and support system and other elements. For example, the hospital can set up the “Spiritual Care Excellence Award” to recognize the group of nurses who continue to show excellent spiritual care behavior, so as to further highlight the value of these comprehensive initiatives. By integrating these three elements into a unified framework, nursing management can build a sustainable organizational environment that addresses both the motivational and ability barriers to spiritual care identified in this study. It can systematically promote spiritual care practices. In the end, this will improve the well-being of both nurses and patients. Additionally, these strategies should address both motivational and capacity barriers and ensure that nurses are willing and able to provide spiritual care with the hospital’s support. Moreover, hospital administrators should mandate spiritual leadership, career calling development, and empathy training in nursing guidelines to meet the unmet spiritual needs of patients and improve the nursing experience of nurses.

Finally, these research results can provide guidance for health policy and governance. Given that the WHO has regarded spirituality as a core dimension of health, hospital managers should formally incorporate spiritual care into a key component of high-quality care. This kind of spiritual care must be incorporated into the practice standards and quality assessment system. For Chinese public hospitals, this evidence supports the shift from a disease-centered to patient-centered medical model.

### 4.3. Limitations and Future Research

There are some limitations of this study that need to be explained. First, this study used convenience and snowball sampling, a non-probability sampling method. While this approach is feasible and widely accepted for recruiting hard-to-reach clinical nurses [47,50], it carries inherent limitations that require careful interpretive caution. Specifically, it may introduce sample composition bias, including potential overrepresentation of nurses with stronger social networks or more favorable work perceptions [69]. We implemented multi-regional sampling, strict eligibility criteria, and achieved a 76.9% response rate to mitigate this bias [53], and our single-source design avoids the fabricated informant ratings common in multisource snowball studies [49,69]. However, these mitigation measures cannot fully eliminate the fundamental limitations of non-probability sampling. Our findings should therefore be interpreted with appropriate caution and generalized only to similar clinical contexts, not to the entire nursing population in China. To address this limitation, future research should combine snowball sampling with probability-based methods to improve sample representativeness [69], such as stratified sampling across hospital levels, regions, and tenure groups.

Second, the data were single-source and self-reported, which raises the possibility of common method bias [59]. While we conducted statistical analyses to assess this issue, we acknowledge that these tests alone cannot fully eliminate the inherent limitations of self-reported data. Future research should collect multi-source data (e.g., nurses’ rating of spiritual leadership, patients’ rating of nurses’ spiritual care) to reduce common method bias and provide a more comprehensive assessment of the constructs.

Third, our survey design was correlational in nature, which may raise concerns about reversed causality (e.g., nurses who provide more spiritual care may have an increased career calling experience). Moreover, reciprocal relationships are theoretically plausible. For example, nurses who consistently engage in spiritual care may experience a stronger sense of career calling, which in turn may make them more likely to perceive and appreciate spiritual leadership behaviors (e.g., altruistic love) from their supervisors. Similarly, nurses with higher levels of empathy may be more attuned to their leaders’ altruistic actions and thus report higher levels of perceived spiritual leadership. To address the limitations of cross-sectional designs and strengthen causal inference, future studies should implement longitudinal research designs to examine the temporal relationships between the variables. This will help clarify the direction of causality and test the stability of the hypothesized model.

Fourth, this study did not directly measure or control for organizational level factors such as the nurse-to-patient ratio, workload, organizational culture, and hospital management policies. These factors have been shown to affect nurse well-being and spiritual care [70]. Future research should explicitly incorporate organizational level variables (e.g., workload, organizational climate) into the model to examine their moderating effects and disentangle individual versus organizational factors associated with nurses’ career calling and spiritual care behaviors.

Fifth, this study only focuses on the hospital environment in China, which may limit the applicability of the results. Cultural and institutional differences in spiritual leadership, professional identity and empathetic expression may affect the cross-cultural effectiveness of the model. Future research should repeat experiments in different cultural contexts, such as Western medical systems or other Asian countries with significant differences in social culture and medical institutions. For example, examine how cultural dimensions, such as the nursing team’s power distance climate as the moderating variable, affect and identify boundary conditions for the applicability of spiritual leadership in different healthcare contexts.

Sixth, our model assumes linear relationships between all constructs, but non-linear effects may exist. For example, excessively high levels of spiritual leadership could potentially lead to role overload or emotional exhaustion among nurses, resulting in a U-shaped or inverted U-shaped relationship with spiritual care behavior. Similarly, very high levels of empathy may lead to compassion fatigue, which could reduce rather than enhance spiritual care provision. Future research should examine these potential non-linear relationships to develop a more nuanced understanding of the model.

Seventh, the research results showed that empathy primarily functions as a moderator rather than a mediator or direct correlate in the present model, when controlling for career calling and spiritual leadership. However, this does not limit the possibility that empathy may play different roles in other theoretical frameworks or cultural contexts. Consistent with the literature we cited, prior studies have documented empathy as a direct predictor of spiritual care behavior (e.g., refs. [4,71]). Future cross-cultural comparative studies could explore how cultural values (such as collectivism vs. individualism) shape the role of empathy in spiritual care delivery, providing a more comprehensive understanding of the boundary conditions of our model.

## 5. Conclusions

This study emphasizes that spiritual leadership is crucial to cultivating nurses’ career calling. However, motivation alone does not guarantee practical action. The successful implementation of spiritual care behavior depends on personal ability (empathetic ability) and environmental alignment (person–environment fit). By integrating multiple theoretical perspectives, this study comprehensively explains the gap between motivation and action, and determines that leadership, personal ability and workplace adaptability are important parts of promoting spiritual care in the medical environment. Empirical research emphasizes that it is not only necessary to cultivate meaningful work (career calling), but also to cultivate the skills needed to transform theoretical cognition into clinical practice. By implementing supportive leadership and capacity-building interventions, medical institutions can bridge the gap between spiritual care awareness and practice, thus promoting comprehensive and empathic nursing services to meet the spiritual health needs of patients.

## Figures and Tables

**Table 1:** The demographic and work-related characteristics of participants (N = 323).

Variable	Category	N (%)	χ^2^ (*p*)
Age	≤30 years	113 (34.9)	157.72 (<0.001)
	31–45 years	144 (44.7)	
	≥46 years	66 (20.4)	
Gender	Male	70 (21.7)	103.68 (<0.001)
	Female	253 (78.3)	
Tenure	1–2 years	46 (14.2)	197.04 (<0.001)
	3–5 years	50 (15.5)	
	6–15 years	118 (36.6)	
	16–25 years	56 (17.3)	
	≥26 years	53 (16.2)	
Marital Status	Single	84 (26)	267.13 (<0.001)
	Married	239 (74)	
Monthly Salary	≤2500 Yuan	23 (7.1)	89.34 (<0.001)
	2501–5000 Yuan	77 (23.8)	
	5001–7500 Yuan	109 (33.7)	
	7501–10,000 Yuan	87 (26.9)	
	>10,000 Yuan	27 (8.4)	
Job Satisfaction	Very Dissatisfied	8 (2.5)	223.30 (<0.001)
	Somewhat Dissatisfied	16 (5)	
	Neutral	89 (27.6)	
	Generally Satisfied	155 (48)	
	Very Satisfied	55 (17)	

**Table 2:** Confirmatory Factor Analysis Results.

Model	χ^2^	∆χ^2^	df	RMSEA	CFI	TLI	SRMR
baseline 4-factor model	84.875	—	48	0.049	0.988	0.984	0.027
M1: alternative 3-factor model	558.536	473.661 ***	51	0.176	0.836	0.787	0.111
M2: alternative 3-factor model	679.751	594.876 ***	51	0.195	0.797	0.737	0.142
M3: alternative 3-factor model	714.12	629.245 ***	51	0.201	0.785	0.722	0.149
M4: alternative 3-factor model	566.423	481.548 ***	51	0.177	0.833	0.784	0.092

Note: *** *p* < 0.001. Δχ^2^ = Change in Chi-square; CFI = Comparative Fit Index; TLI = Tucker–Lewis Index; RMSEA = Root Mean Square Error of Approximation; SRMR = Standardized Root Mean Square Residual. Model 1: Spiritual leadership and career calling were combined into one factor; Model 2: Spiritual leadership and spiritual care behavior were combined into one factor; Model 3: Career calling and empathy were combined into one factor; Model 4: Career calling and spiritual care behavior were combined into one factor.

**Table 3:** Correlations and Descriptive Statistics.

	M	SD	1	2	3	4	5	6	7	8	9	10	Cronbach’s α
1. Age	35.78	9.45	—										
2. Gender ^a^	0.78	0.41	−0.02	—									
3. Tenure	13.02	10.12	0.40 **	−0.04	—								
4. Marital Status ^b^	0.74	0.44	0.26 **	−0.01	0.54 **	—							
5. Monthly salary	3.06	1.06	0.05	−0.08	0.24 **	0.11 *	—						
6. Job Satisfaction	3.72	0.89	0.19 **	−0.06	0.31 **	0.19 **	0.04	—					
7. Spiritual Leadership	3.70	0.87	0.08	−0.07	0.06	0.01	−0.14 *	0.45 **	—				0.98
8. Career calling	3.78	0.69	0.11 *	−0.12 *	0.20 **	0.20 **	0.07	0.40 **	0.56 **	—			0.91
9. Spiritual Care Behavior	4.24	0.63	0.04	−0.08	0.14 *	0.16 **	0.15 **	0.31 **	0.44 **	0.52 **	—		0.91
10. Empathy	3.95	0.53	−0.05	−0.01	0.06	−0.02	−0.05	0.15 **	0.22 **	0.13 *	0.32 **	—	0.90

Note: N = 323; ** *p* < 0.01,* *p* < 0.05. Reliability coefficients are reported in the last column. ^a^ 0 = male, 1 = female. ^b^ 0 = single, 1 = married.

**Table 4:** Regression results of process (N = 323).

Variable	Mediation		Moderated Mediation	
	M1 Career Calling b(SE)	M2 Spiritual Care Behavior b(SE)	M3 Career Calling b(SE)	M4 Spiritual Care Behavior b(SE)
Constant	1.53 ***	1.85 ***	−2.26 ***	3.07 ***
Controls				
Age	−0.00 (0.00)	−0.00 (0.00)	−0.00 (0.00)	0.00 (0.00)
Gender	−0.12 (0.08)	−0.01 (0.07)	−0.12 (0.07)	−0.03 (0.06)
Tenure	0.00 (0.00)	−0.00 (0.00)	0.00 (0.00)	−0.00 (0.00)
Marital Status	0.22 ** (0.08)	0.12 (0.08)	0.22 ** (0.08)	0.10 (0.07)
Monthly salary	0.07 * (0.03)	0.12 (0.08)	0.07 * (0.03)	0.11 ** (0.03)
Job Satisfaction	0.10 * (0.04)	0.04 (0.04)	0.10 * (0.04)	0.03 (0.04)
Spiritual Leadership	0.40 *** (0.04)	0.18 (0.04) ***	0.40 *** (0.04)	0.17 *** (0.04)
Career calling		0.31 (0.05) ***		0.31 *** (0.05)
Empathy				0.27 *** (0.05)
Career calling × Empathy				0.43 *** (0.08)
*R* ^2^	0.38 ***	0.33 ***	0.38 ***	0.44 ***

Note: * *p* < 0.05, ** *p* < 0.01. *** *p* < 0.001. The values in the table are path estimates from the estimated model. Unstandardized regression coefficients are reported. Bootstrap sample size = 5000.

**Table 5:** Regression Results for Conditional Indirect Effect.

Predictor	Boot Indirect Effect	Boot SE	[Boot LL 95%CI–Boot UL 95% CI]
Simple Mediation Effect			
	0.14	0.03	[0.09, 0.19]
Moderated Mediation Effect			
High (+1SD)	0.21	0.03	[0.16, 0.27]
Mean	0.12	0.03	[0.08, 0.18]
Low(−1SD)	0.03	0.03	[−0.01, 0.08]
Index of moderated mediation (difference between conditional indirect effects)			
Empathy	0.17	0.03	[0.11, 0.23]

Note. N = 323. Unstandardized regression coefficients are reported. Bootstrap sample size = 5000. LL = lower limit; CI = confidence interval; UL = upper limit.

## Data Availability

Data are not available due to ethical and confidentiality restrictions.

## Abbreviations

The following abbreviations are used in this manuscript:

CFA	Confirmatory Factor Analysis
CFI	Comparative Fit Index
ICU	Intensive Care Unit
RMSEA	Root Mean Square Error of Approximation
SCB	spiritual care behavior
SDT	Self-Determination Theory
SRMR	Standardized Root Mean Square Residual
TLI	Tucker–Lewis Index
WHO	World Health Organization
